# Plane of nutrition affects the phylogenetic diversity and relative abundance of transcriptionally active methanogens in the bovine rumen

**DOI:** 10.1038/s41598-017-13013-y

**Published:** 2017-10-12

**Authors:** Emily McGovern, Matthew S. McCabe, Paul Cormican, Milka Popova, Kate Keogh, Alan K. Kelly, David A. Kenny, Sinead M. Waters

**Affiliations:** 1 Teagasc, Animal and Bioscience Research Department, Animal and Grassland Research and Innovation Centre, Teagasc, Grange, Dunsany, County Meath Ireland; 2 UCD, College of Health and Agricultural Sciences, University College Dublin Belfield, Dublin 4, Ireland; 3 UMR1213 Herbivores, INRA, VetAgro Sup, Clermont Université, Université de Lyon, 63122 Saint Genès-Champanelle, France

## Abstract

Methane generated during enteric fermentation in ruminant livestock species is a major contributor to global anthropogenic greenhouse gas emissions. A period of moderate feed restriction followed by *ad libitum* access to feed is widely applied in cattle management to exploit the animal’s compensatory growth potential and reduce feed costs. In the present study, we utilised microbial RNA from rumen digesta samples to assess the phylogenetic diversity of transcriptionally active methanogens from feed-restricted and non-restricted animals. To determine the contribution of different rumen methanogens to methanogenesis during dietary restriction of cattle, we conducted high-throughput *mcrA* cDNA amplicon sequencing on an Illumina MiSeq and analysed both the abundance and phylogenetic origin of different *mcrA* cDNA sequences. When compared to their unrestricted contemporaries, in feed-restricted animals, the methanogenic activity, based on *mcrA* transcript abundance, of *Methanobrevibacter gottschalkii* clade increased while the methanogenic activity of the *Methanobrevibacter ruminantium* clade and members of the Methanomassiliicoccaceae family decreased. This study shows that the quantity of feed consumed can evoke large effects on the composition of methanogenically active species in the rumen of cattle. These data potentially have major implications for targeted CH_4_ mitigation approaches such as anti-methanogen vaccines and/or tailored dietary management.

## Introduction

The 2015 UNFCCC Paris agreement aims to pursue efforts to limit the increase in global warming to 1.5 °C^1^ above temperatures prevailing during the pre-industrialisation era^1^. Globally, agriculture, forestry and other land use account for about 24% of annual greenhouse gas (GHG) emissions^2^. These include direct emissions from the cultivation of crops and livestock and also as a result of deforestation^3^. Enteric methane (CH_4_) emissions from the livestock sector is the single largest contributor to global CH_4_ emissions (40%)^3^.

Most of the variation in the composition of the rumen microbiota is a direct result of feed composition and quantity consumed^4–6^. A period of feed-restriction followed by *ad libitum* feeding is widely used in livestock management for the beef industry to reduce feed costs^7^. Following a period of dietary restriction cattle typically undergo accelerated growth referred to as compensatory growth^8^, upon re-alimentation. Feed restricted animals have been previously shown to have slower passage rate of feed through the rumen^9,10^ which has previously been correlated with increased CH_4_ emissions per unit of feed from ruminants^9,11,12^. We previously found that feed-restricted animals had increased relative abundance of 16S DNA from the *Methanobrevibacter gottschalkii* clade^5^. This observation was positively correlated with a dramatic decrease (from 30% to less than 1%) in the relative abundance of a single bacterial 16S DNA sequence which could only be identified confidently as an uncultured Proteobacteria species and appeared to be related to the family Succinivibrionaceae^5^. As some Succinivibrionaceae species utilise hydrogen^13^ we hypothesized that the aforementioned putative Succinivibrionaceae species was in competition for substrate, possibly hydrogen, with the *Methanobrevibacter gottschalkii* clade^5^ in feed restricted animals. This was consistent with a decrease in ruminal propionate and attendant increased acetate:propionate (A:P) ratio, an indication of greater methanogenesis in these animals^5^. Following two months of re-alimentation, the volatile fatty acid ratios of ruminal digesta from previously feed restricted animals mirrored that of their non-restricted contemporaries, while a reversal in the relative abundance of 16S DNA from the *Methanobrevibacter gottschalkii* clade and the putative Succinivibrionaceae 16S DNA^5^ was also observed.

It is still not clear if analysis of 16S DNA accurately reflects the metabolic activity of rumen bacteria and archaea as metabolically inactive and dead bacteria retain large amounts of DNA^14^. We were therefore interested in determining which ruminal methanogens showed the highest methanogenic activity in cattle undergoing feed restriction followed by re-alimentation and compensatory growth. To do this we employed high-throughput amplicon sequencing of *mcrA* gene cDNA. Methyl co-enzyme reductase (MCR) is a multi-subunit enzyme of methanogenic archaea which catalyses the final step in CH_4_ formation and the initial step in anaerobic CH_4_ oxidation^15^. It reduces methyl-coenzyme M (CH3-S-CoM) by 7-mercaptoheptanoylthreonine phosphate (H-S-HTP)^16^. The MCR subunits are coded for by the *mcr* gene operon which usually comprises the genes *mcrA, B, C, D* and *G*. The *mcrA* gene codes for subunit A of MCR and can be used both as a phylogenetic marker for the identification of archaea and, as *mcrA* transcript abundance is positively correlated with methane production in peat soil^17^, is a quantitative marker for methane production. The objective of this study was to determine which methanogens were contributing to methanogenesis in the bovine rumen during periods of dietary restriction. To our knowledge this is the first time that high-throughput sequencing of *mcrA* cDNA amplicon libraries has been used to explore the diversity of metabolically active methanogens present in the rumen. This may be useful as a convenient and inexpensive method for use in large numbers of animals to answer the, as yet unanswered question, ‘which methanogens are making the greatest relative contribution to ruminal methane production’?

## Results

### Animal Performance

Data on the performance of the animals from which ruminal digesta was harvested for this study, have been reported previously by Keogh *et al*.^8^. Abbreviations differ in previous publication due to different scientific objectives and for ease of comparison. A and AA refer to ADLIB from period one and two respectively, R and RA refer to RES period one and period two respectively^8^. Briefly, dry matter intake (DMI) (kg/d) for each animal group was A = 12.80 ± 0.43, R = 5.44 ± 0.17, AA = 12.22 ± 0.98 and RA = 11.8 ± 0.97. The average daily increase in body weight (kg) for each group was, A = 1.93 ± 0.15, R = 0.62 ± 0.07, AA = 1.4 ± 0.30 and RA = 2.5 ± 0.52. The average body weight of the animals at slaughter (kg) for each group was, A = 631.8 ± 41.6, R = 466.1 ± 44.6, AA = 667.1 ± 56.0, and RA = 575.5 ± 36.9. Body weight of R animals was less (P < 0.05) than that of A animals and similarly body weight of RA was less (P < 0.05) than that of AA animals.

Weight of both the full and empty reticulorumen complex was recorded at slaughter^8^. While the weight of the reticulorumen was lower for R animals in comparison to the other treatments, there was no difference in the weight of digesta between the treatment groups, potentially reflecting the slower ruminal passage of digesta in R animals.

### Phylogenetic Analysis

**Figure 1:**
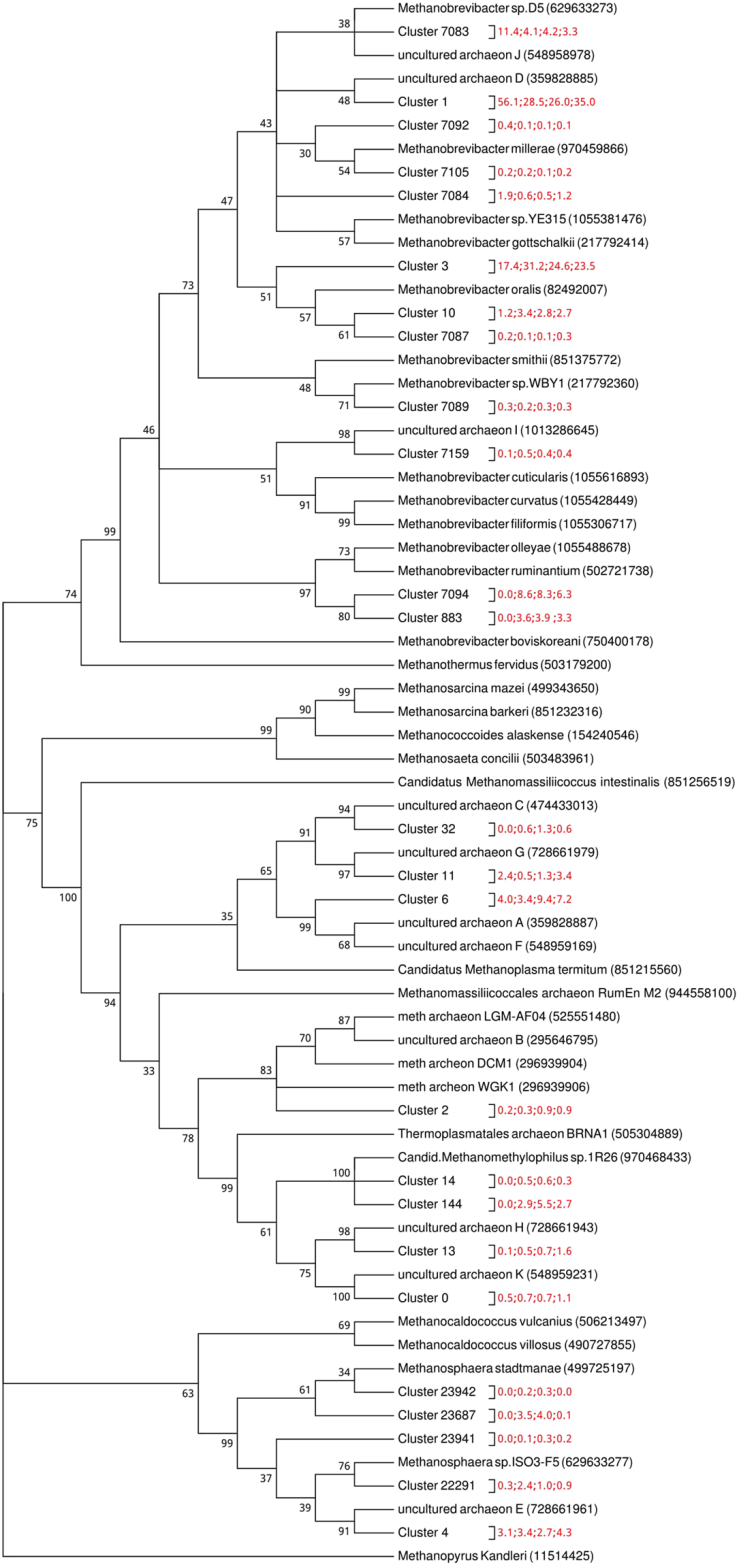
The evolutionary history which approximately identifies the phylogeny of clustered sequences was inferred using the Maximum Parsimony method. The percentage of replicate trees in which the associated taxa clustered together in the bootstrap test (500 replicates) are shown next to the branches. Branches with less than 30% support were collapsed. The MP tree was obtained using the Subtree-Pruning-Regrafting (SPR) algorithm with search level 1 in which the initial trees were obtained by the random addition of sequences (10 replicates). All positions with less than 85% site coverage were eliminated. There were a total of 157 positions in the final dataset. The 4 numbers in red separated by a “;” after each cluster represent the average representation of this cluster in R, A, RA and AA groups respectively.

A total of 19 from 25 MCRA clusters were differentially represented amongst the four treatment groups (Fig. 1). Methanobacteriaceae was the dominant family identified from the phylogenetic analysis. Twelve MCRA clusters were identified to be the most closely related to the genus *Methanobrevibacter* (MCRA cluster 1, 3, 10, 883, 7083, 7084, 7087, 7089, 7092, 7094, 7105 and 7159) and five MCRA clusters were grouped with another genus from the Methanobacteriaceae family; *Methanosphaera* (MCRA Cluster 4, 22291, 23687, 23941, 23942). The remaining eight MCRA clusters were identified to be related to the Methanomassiliicoccaceae family (MCRA cluster 0, 2, 6, 11, 13, 14, 32, and 144). Of these twelve MCRA clusters with *Methanobrevibacter* lineage, MCRA cluster: 1, 7083, 7084, 7092 and 7105 grouped with known methanogens from the *Methanobrevibacter gottschalkii clade*. Two MCRA clusters: 883 and 7094, assembled closely with members of the *Methanobrevibacter ruminantium* clade.

Relative abundance of MCRA amino acid sequences (translated from *mcrA* cDNA) indicated that the family Methanobacteriaceae had the highest methanogenic activity in the rumen of all bulls sampled regardless of treatment (Table 1). There was no difference in Methanobacteriaceae (P > 0.05) between the R and A groups although relative abundance of translated *mcrA* transcripts from this family were increased (P < 0.001) in group R compared to groups RA and AA.

**Table 1:** Mean relative abundances for the four treatment groups of clusters identified from maximum parsimony tree at family and genus level.

	MCRA Cluster Identification	Treatment				*P* value
		R	A	RA	AA	
Family	Methanobacteriaceae	92.7^a^	89.4^a,b^	81.9^b^	79.0^b,c^	<0.001
Family	Methanomassiliicoccaceae	7.3^a^	10.7^a,b^	18.1^b,c^	21.1^c^	<0.001
Genus	*Methanobrevibacter*	89.2^a^	79.0^b^	74.8^b^	72.1^b^	<0.001
	*M*. *ruminantium clade*	0.1^a^	14.2^b^	11.6^b^	10.3^b^	<0.0001
	*M*. *gottschalkii clade*	70.0^a^	25.3^b^	36.1^b^	32.6^b^	<0.0001
Genus	*Methanosphaera*	3.5^a^	10.4^a^	7.1^a^	6.9^a^	NS

*P*-values are FDR corrected and derived from Wilcoxon pairwise comparisons of MCRA clusters between different treatment groups.

There was no difference in the relative abundance of MCRA clusters assigned to *Methanosphaera* (P > 0.05), a genus of the Methanobacteriaceae family, between any of the four treatment groups. The major changes in relative abundance of translated *mcrA* transcripts were attributed to the dominant genus *Methanobrevibacter*. Relative abundance of *Methanobrevibacter* was elevated in group R in comparison to groups A, AA and RA (P < 0.001). The abundance of *mcrA* transcripts assigned to the *M*. *gottschalkii* clade was increased in group R relative to all other treatments groups (P < 0.0001) whereas the *mcrA* transcripts assigned to the *M*. *ruminantium* clade were dramatically reduced in group R compared to groups A, AA and RA (P < 0.0001).

Methanomassiliicoccaceae was not statistically significantly different between treatment groups A and R. However, it did differ between treatment groups A and R and between treatment groups AA and RA (P < 0.001) (Table 1).

### *Methanobrevibacter ruminantium* clade

MCRA clusters 7094 and 883 were the only clusters that were assigned to the *M*. *ruminantium* clade (Fig. 1) and both were (P < 0.0001) reduced in the restricted group R in comparison to groups A, AA and RA.

### *Methanobrevibacter gottschalkii* clade

Five MCRA clusters (1, 7083, 7084, 7092 and 7105) (Table 2) were assigned to the *M*. *gottschalkii* clade. Four of these MCRA clusters (1, 7083, 7084 and 7092) were increased (P > 0.0001) in group R relative to groups A, AA and RA. MCRA cluster 1 is the largest cluster of the dataset as the majority of translated *mcrA* cDNA sequences were assigned to this cluster. The relative abundance of this MCRA cluster was increased by approximately 2-fold in the feed restricted animals.

**Table 2:** Mean relative abundances of the 25 MCRA clusters for the four treatment groups.

MCRA Cluster	Treatment				*P*-value
	R	A	RA	AA	
0	0.5^a^	0.8^a^	1.0^a^	0.9^a^	NS
1	56.1^a^	21.0^b^	30.7^b^	28.6^b^	<0.01
2	0.2^a^	0.3^a,b^	0.9^b,c^	0.9^c^	<0.001
3	17.4^a^	34.7^a^	23.6^a^	25.3^a^	NS
4	3.1^a^	3.2^a^	3.4^a^	3.6^a^	NS
6	4.0^a^	4.0^a^	8.0^a^	9.0^a^	NS
10	1.2^a^	3.9^b^	2.7^a,b^	2.9^a,b^	<0.05
11	2.4^a^	0.5^b^	2.0^a^	2.8^a^	<0.01
13	0.1^a^	0.5^a,b^	0.8^b^	1.6^b^	<0.001
14	0.0^a^	0.6^b^	0.4^b^	0.5^b^	<0.001
32	0.0^a^	0.7^b^	1.2^b^	0.8^b^	<0.001
144	0.0^a^	3.4^b^	3.8^b^	4.63^b^	<0.001
883	0.0^a^	4.1^b^	3.8^b^	3.5^b^	<0.001
7083	11.5^a^	3.6^b^	4.6^b^	2.7^b^	<0.001
7084	1.9^a^	0.3^b^	0.7^b^	1^a,b^	<0.01
7087	0.2^a^	0.1^b^	0.2^a,b^	0.2^a^	<0.01
7089	0.3^a^	0.2^a^	0.2^a^	0.4^a^	NS
7092	0.4^a^	0.1^b^	0.1^b^	0.1^b^	<0.001
7094	0.0^a^	10.0^b^	7.8^b^	6.8^b^	<0.001
7105	0.2^a^	0.2^a^	0.1^a^	0.3^a^	NS
7159	0.1^a^	0.6^b^	0.5^a,b^	0.4^a,b^	<0.05
22291	0.3^a^	2.8^b^	1.1^bc^	0.9^c^	<0.001
23687	0.0^a^	4.1^b^	2.3^b^	1.8^b^	<0.01
23941	0.0^a^	0.1^b^	0.1^b^	0.4^b^	<0.01
23942	0.0^a^	0.2^b^	0.2^b^	0.1^a,b^	<0.01

*P*-values are FDR corrected and derived from Wilcoxon Pairwise comparisons of MCRA clusters between different treatment groups.

### Methanomassiliicoccaceae

There was no difference between treatments in the overall relative abundance of MCRA clusters assigned to the family Methanomassiliicoccaceae (Table 1). However, the relative abundance of four individual MCRA clusters (13, 14, 32 and 144) within this family was reduced (P < 0.001) in treatment group R compared to groups A, AA and RA (Table 2).

### Ordination of Diversity of MCRA clusters

**Figure 2:**
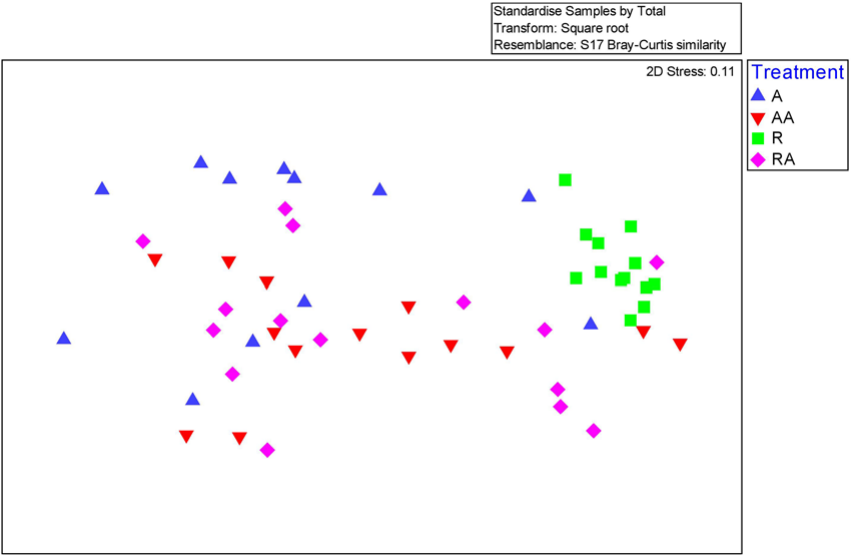
nMDS plot indicating similarity of metabolically active methanogens between treatments. Distance between samples, based on similarity of cluster composition (similarity 97%) of each sample calculated using Bray-curtis similarity index and plotted using non-metric multidimensional scaling (nMDS). Each point represents a different sample plotted according to their cluster conformation and abundance (stress value = 0.1). A greater distance between two points infers a lower similarity between them, whereas samples composed of similar MCRA clusters will group closer together.

**Figure 3:**
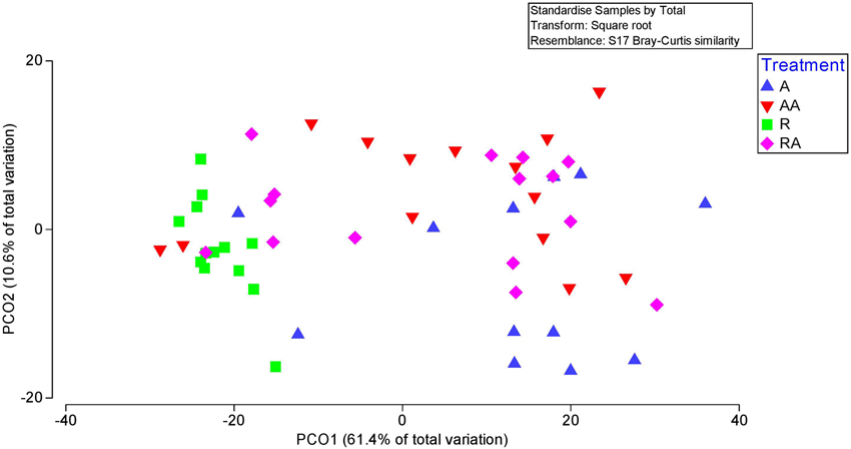
Principal coordinate analysis (PCoA) of the relative abundances of clusters. PCoA was used to compare variation of relative cluster abundances within the liquid rumen samples from treatment A, AA, R and RA.

**Figure 4:**
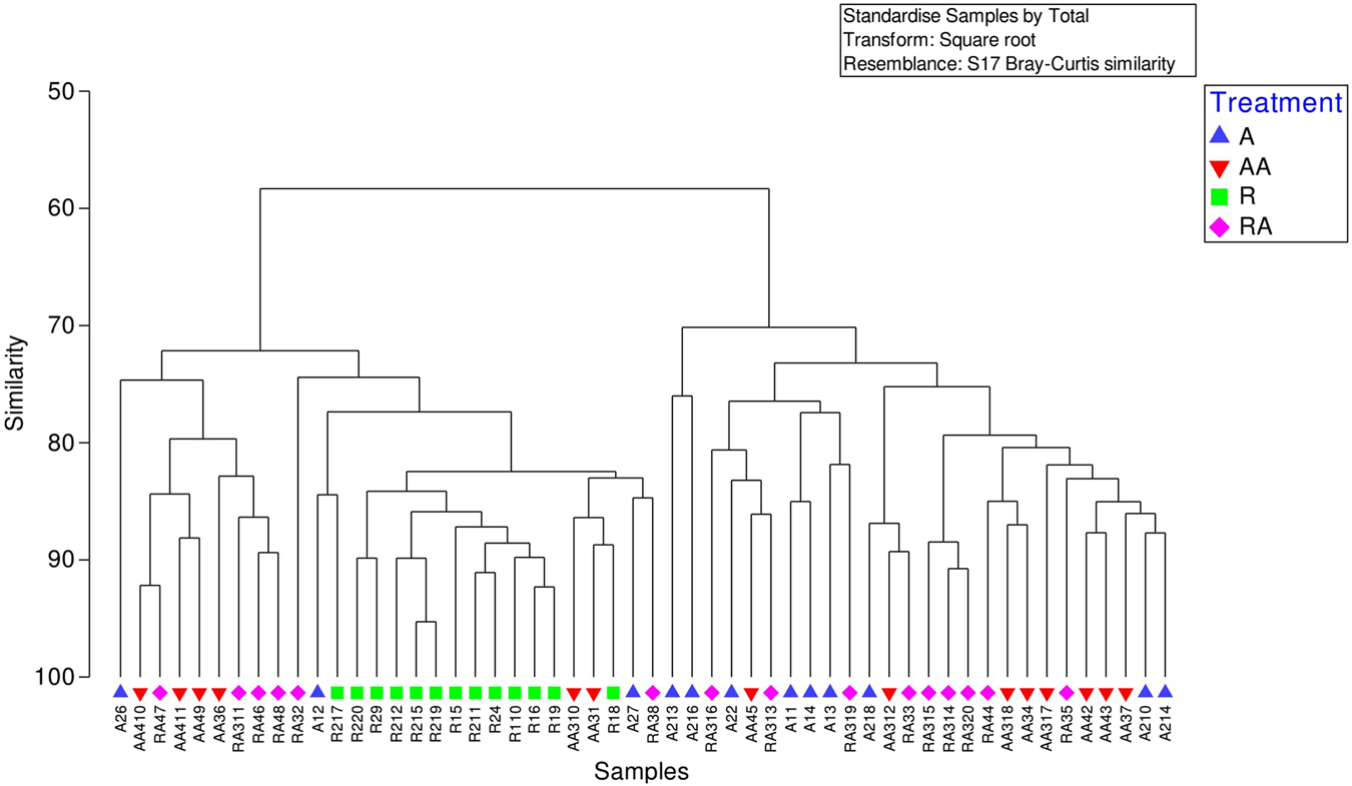
Dendrogram of Bray-Curtis similarity of MCRA clusters in treatment groups; A, AA, R and RA.

nMDS (Fig. 2) and PCoA (Fig. 3) plots and dendrogram analysis (Fig. 4) were used to assess the similarities and diversity of populations of MCRA clusters within the four treatment groups. These show a tight cluster of points representing the restricted animals which is separate from most of the points representing animals from the *ad libitium* (A, AA and RA) treatment groups. This indicates that methane was being generated by different methanogen species in the feed-restricted animals than in the *ad libitum* fed animals. These cluster analyses also suggests that the methanogenically active methanogens were more similar between feed-restricted animals than between *ad libitum* animals.

**Figure 5:**
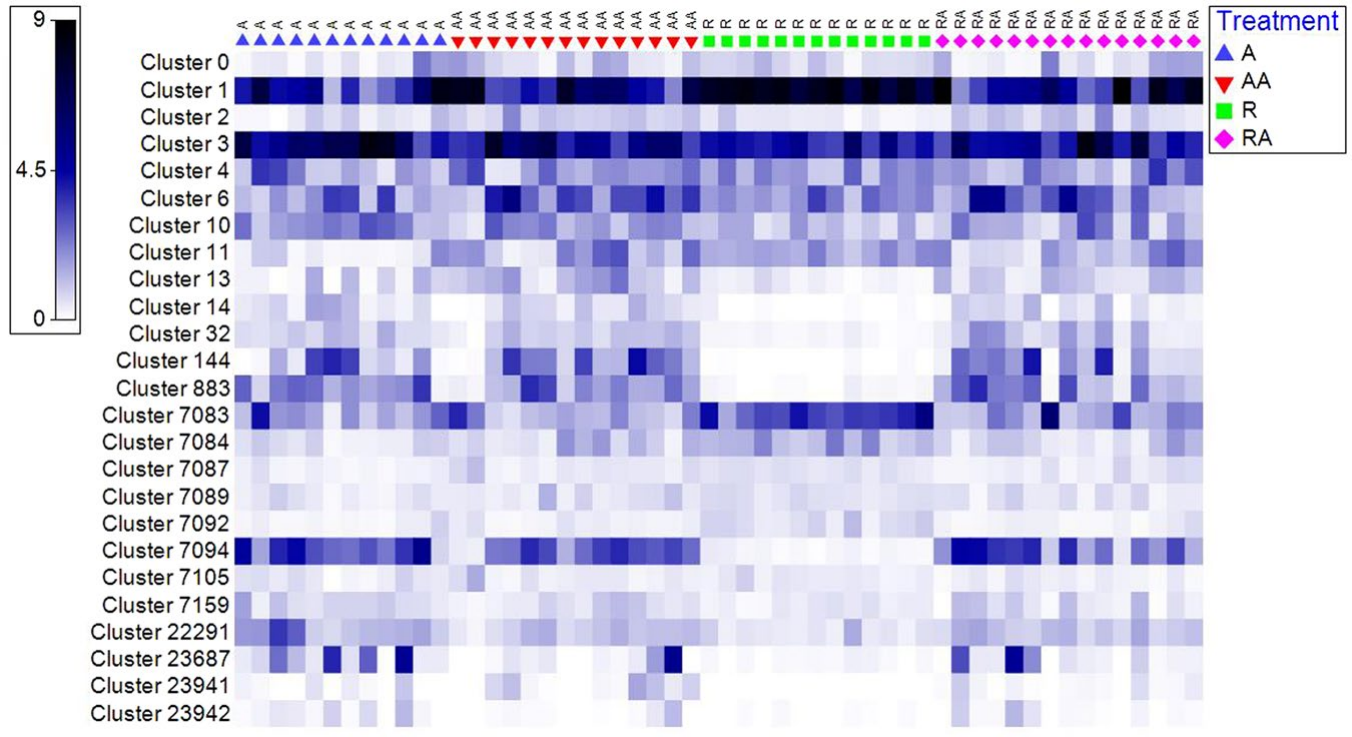
Square root transformed relative abundance heat map (white = absence, dark blue = high) of MCRA clusters in treatment groups; A, AA, R and RA.

Diversity matrices also showed a reduction in diversity of MCRA clusters in treatment group R relative to the three *ad libitum* treatment groups (Table 3). This was apparent in the heatmap (Fig. 5). A distinct pattern of MCRA clusters representing active methanogens that are specific to the R bulls was observed. The heatmap highlights the relative increase in representation and increased homogeneity between samples of the MCRA clusters 1, 7083, 7084, 7092 of the *M*. *gottschalkii* clade in restricted animals in comparison to treatment groups A, AA and RA. The relative under representation and homogeneity between samples of MCRA cluster 883 and 7094 of the *M*. *ruminantium* clade and MCRA cluster 13, 14, 32 and 144 of the Methanomassiliicoccaceae family in treatment group R in comparison to treatment groups A, AA, RA was also evident from the heatmap. MCRA cluster 3 of *Methanobrevibacter* lineage was not significantly different between treatment groups, despite a numerical difference in R group bulls (Table 2). This cluster had the second greatest relative methanogenic activity regardless of treatment and this activity is consistent across treatment. This can be visualised from the heatmap (Fig. 5).

**Table 3:** Effect of dietary restriction and *ad libitum* feeding, pre and post restriction, on *alpha* diversity, species richness and presence of MCRA clusters in treatment groups; A, AA, R and RA.

	Treatment				*P* value
	R	A	RA	AA	
MCRA Clusters	21^a^	24^b^	24^b^	24^b^	<0.0001
Richness	1.41^a^	2.22^b^	2.09^b^	2.05^b^	<0.0001
Shannon	1.41^a^	1.83^a,b^	1.85^b^	1.96^b^	<0.0001
Simpson	0.62^a^	0.74^b^	0.72^a,b^	0.76^b^	<0.01

*P*-values are derived using an one-way ANOVA with a Tukey HSD test was being used to assess the differences between treatments.

## Discussion

We previously reported, using high-throughput 16S DNA amplicon sequencing, that a period of moderate feed restriction led to greater abundance of *M*. *gottschalkii* clade in the ruminal liquor of moderately feed restricted animals with an inverse relationship to a putative Succinivibrionaceae species^5^. Results from the study^5^ show that both ruminal fractions, i.e. solid and liquid, exhibited similar community diversity, however, differences linked to feeding regimes were amplified in the rumen liquid. This informed the decision to go forward with detailed examination of the microbial RNA of the rumen liquid fraction in order to address the main research objective to identify which methanogens dominate methanogenesis during periods of bovine dietary restriction.

Our approach utilised *mcrA* cDNA amplicon sequencing to determine the abundance of *mcrA* transcripts and their phylogenetic origin to identify the predominant methane generating species under different feed treatments. High-throughput *mcrA* amplicon sequencing, appears to have potential as a convenient and inexpensive tool for the identification of methane producing archaea and to assess their potential contribution to methane production in a large number of animals. There are two isoenzymes of MCR, denoted MCRI, which is encoded by operon *mcrABCDG* and MCR II, encoded by operon *mrtABDG* ^16^. The different isoenyzme systems allow methanogens to be supported by different growth environments^18^. The *mcrA* gene is contained in both enzyme operons hence making it a useful molecular marker for methanogen activity in different environments.

The RNA gene expression work described here partly supports the results of our 16S DNA amplicon sequencing. Methanobacteriaceae and Methanomassiliicoccaceae were found to be the dominant families in all treatment groups both at the RNA and DNA level. *Methanobrevibacter* was both the dominant methanogenic genus present in the 16S DNA study and produced the most *mcrA* transcripts relative to the other methanogen genera detected in the current study. The 16S amplicon sequencing analysis that we previously reported on these animals showed that relative abundance of the *M*. *gottschalkii* clade increased in bulls fed a restricted diet in comparison to the cattle fed an *ad libitum* diet^5^. The present study shows that this increase also occurred at the RNA level, indicating that feed restriction favours members of this clade. However, whereas our 16S DNA amplicon study on these animals showed that members of the *M*. *ruminantium* clade remained unchanged between treatments, our *mcrA* cDNA amplicon study shows a dramatic decrease in abundance in restricted animals. There are at least two possible explanations for this discrepancy. The first of these is that 16S DNA amplicon sequencing detects the presence or absence of bacteria and/or archaea, but does not give direct information on the activities and physiological states of microorganisms in samples^14^. The *M*. *ruminantium* clade detected in our 16S DNA amplicon study may have been present but not generating methane, while our *mcrA* cDNA amplicon data most likely provides a more accurate picture of which species are generating methane in the rumen. Secondly, our 16S DNA amplicon sequencing simultaneously targeted bacteria and archaea so it was possible to determine the abundance of archaea relative to bacteria. The *mcrA* primers, however, only show abundance of archaea relative to other archaea so the large increase in clusters in MCRA clusters in the *M*. *gottschalkii* clade would make it appear as though there was a decrease in other MCRA clusters. To resolve this, a further study is required in which an exogenous control RNA sequence is added at the start of RNA extraction to obtain an absolute rather than relative measure of *mcrA* transcript abundance. Alternatively, RNA-Seq analysis, for example, would permit the identification of *mcrA* transcripts relative to all other transcripts. Relative abundance of Methanomassiliicoccaceae did not significantly differ between treatment groups at either the DNA or RNA level.

The phylogenetic assembly and relative abundance of *mcrA* cDNA found in this study are in agreement with the current literature on rumen composition derived from DNA based investigations^11,19,20^. This includes an international study^19^, which found that there is consistency in ruminant bacterial and archaeal communities regardless of species, diet and geographical location. Members of the *M*. *gottschalkii* and *M*. *ruminantium* clades were the two largest methanogenic groups in the rumen, accounting for approximately 74% of the total archaeal population^19^. Our *mcrA* cDNA results were consistent with this regardless of treatment. The two other dominant methanogen groups were *Methanosphaera* and Methanomassiliicoccaceae. In total, these four groups account for 89.2% of the total rumen archaeal population.

Significantly, we have identified, using alpha diversity analysis, that feed restriction has a dramatic effect on species which generate methane in the rumen. Dietary restriction appears to provide a niche environment for specific methanogenic groups and removes the necessary environment required to sustain a more diverse archaeal community. High feed intake was previously associated with increased passage rate^9^ and constant substrate supply to rumen microbes, ensuring diverse substrate to the rumen methanogen population. In our study, the microbiota of the restricted bulls probably resides in a different ecosystem to the *ad libitum* groups due to the smaller rumen size and reduced feed availability^8^. MCRA cluster 3, identified to be of the *Methanobrevibacter* genus, showed the second greatest methanogenic activity in all treatment groups. This indicates that dietary management inferred no bias toward the methanogenic activity of this *Methanobrevibacter* cluster. Feed-restriction did however, affect other clusters within the three major methanogenic groups active in the rumen. The relative abundance of MCRA clusters that were most closely related to the *M*. *ruminantium* clade and Methanomassiliicoccaceae were decreased in the rumen of feed restricted bulls, while the relative abundance of MRCA clusters of the *Mbr*. *gottschalkii* clade was increased.

*M*. *ruminantium* M1 possesses *mcrI* ^21^ but does not possess the *mcrII* gene system. However, another known member of the *M*. *ruminantium* clade, *M*. *olleyae* possess *mcrII* so it is unclear if the lack of *mcrI* is typical of members of the *M*. *ruminantium* clade^22^. If it is assumed that the clusters 7094 and 883 of the *M*. *ruminantium* clade, identified in this current study, possess a similar *mcrI* system to *M*. *ruminantium* M1, then this could explain the reduction in this clade in the feed restricted animals. Expression of *mcrI* and *mcrII* are controlled preferentially on the levels of substrate available to methanogens present^18^. Low and high substrate concentrations for methanogens upregulate the expression of *mcrI* and *mcrII* respectively^18^. Reduced feed intake is correlated with a reduction in apparent digestibility despite an increase in particle retention time^10^. There was increased bacterial alpha diversity in the restricted animals and increased time for microbial fermentation, along with assumed decreased digestibility. Therefore, it was possible that there was a steady state of substrate available to methanogens present in R animals in comparison to animals fed *ad libitum* ^9,10^. In animals fed *ad libitum* feed, there is typically an increase in digestibility leading to rapid substrate (H_2_) availability to methanogens, initially^9^. This has a limiting effect on H_2_ production from ruminal bacteria, as at a high H_2_ concentration, H_2_ producing pathways are less thermodynamically favourable and thus pathways that utilise H_2_ are favoured, such as propionate production^9^. We hypothesise that due to the reduction in feed intake in restricted animals and the reduced degradability, rumen H_2_ concentrations never reach the threshold concentration that was required for the negative feedback to be initiated. Steady substrate availability may have shifted expression from the *mcrI* isoenzyme in favour of *mcrII*, therefore decreasing the activity of members of the *M*. *ruminantium* clade. *M*. *ruminantium* clade members only possess *mcrI* therefore this increased the activity of members of the *gottschalkii* clade, where all members contain both versions of the isoenzyme. The methanogens active in the restricted group also were contained in a rumen with reduced volume in comparison to methanogens active in the *ad libitum* treatment groups^8^. This may contribute to the steady supply of H_2_ present in the rumen and why it is sufficient to switch activity predominately to *mcrII*. In the rumen of animals fed *ad libitum*, the *Mbr*. *ruminantium* clade was active. This may be due to variation in the amount of H_2_ present in the rumen at different times of the day, as members of this clade are active when supply of H_2_ was low.

The succinate producing bacterial family Succinivibrionaceae was significantly increased in animals fed an *ad libitum* diet and virtually absent in animals fed a restricted diet^5^. This bacterial family utilises H_2_ to produce succinate which is rapidly converted to propionate in the rumen^23^. The *ad libitum* groups all had increased propionate in their rumens in comparison to the restricted group^5^. This provides further evidence that in *ad libitum* animals alternative pathways for H_2_ utilisation are favoured^9^, while H_2_ was below the threshold level for alternative pathways to be prioritised in restricted animals and therefore utilised for CH_4_ production^24^ by a less diverse group of methanogen with the majority made up of the *M*. *gottschalkii* clade.

Four MCRA clusters identified to be from the Methanomassiliicoccaceae family were significantly less methanogenically active in the restricted treatment group in comparison to the *ad libitum* treatment groups. The Methanomassiliicoccaceae family are obligate H_2_-dependent methylotrophs, utilising methyl groups from methanol and methylamines (mono-, di-, and tri-methylamine) and methyl thiols for the production of methyl coenzyme^25^. It has previously been hypothesised that there is a positive association between Methanomassiliicoccaceae and Succinivibrionaceae^19^. Succinivibrionaceae degrade pectin^26^ to produce methanol, which is a substrate required for the growth of Methanomassiliicoccaceae^27^. Therefore, dietary restriction may also have an influence on methylotrophic methanogens which utilise by-products of the microbial fermentation other than hydrogen, such as methylamines. This reduces the diversity of the active methanogen population.

In conclusion we sequenced *mcrA* cDNA amplicon libraries to assess the abundance and diversity *mcrA* cDNA sequences present in the bovine rumen of animals undergoing dietary restriction and subsequent compensatory growth. This validated previous 16S DNA based data from our group and showed the most abundant methanogens were indeed the most methanogenically active. The study identifies a clear cohort of active methanogens in an environment where feed intake was restricted and methanogen substrate supply was limited but stable, when compared with when substrate was unrestricted. The majority of methanogens were hydrogentrophic and we hypothesise that H_2_ concentration in the rumen was the main driver of methanogenic activity in the rumen. We found that under the condition of dietary restriction, where CH_4_ production was predicted to be increased, relative abundance of the *M*. *gottschalkii* clade increased, while members of the *M*. *ruminantium* clade showed a dramatic decrease. Relative abundance of members of the Methanomassiliicoccaceae family, which are H_2_ dependant methyltrophs, were also reduced in diet restricted bulls, as H_2_ concentrations are regulated by passage rate. During the *ad libitum* feeding period we predicted that ruminal H_2_ concentration was increased, directing H_2_ utilisation away from CH_4_ production in favour of other H_2_ utilising pathways, such as succinate and propionate production pathways. In these pathways H_2_ was used endogenously in the microbial cell rather than accumulating in the rumen. This contributes to a diverse methanogenic population that can survive at a variety of H_2_ concentrations and also utilises by-products of the alternative H_2_ utilising pathways such as methylamines. Restricted animals had a slower passage rate and a lower rate of digestibility within the rumen, providing a stable H_2_ supply that is directed towards CH_4_ production, giving rise to a low diversity population of active methanogens. This study therefore implies that a common livestock management system used to reduce feed costs may be contributing to increased ruminant CH_4_ production. Research may be able to identify targeted CH_4_ mitigation approaches such as anti-methanogen vaccines, dietary strategies and probiotics and therefore warrant further exploration.

## Methods

### Animal model

All procedures involving animals were approved by the University College Dublin, Animal Research Ethics Committee and licensed by the Irish Department of Health and Children in accordance with the European Community Directive 86/609/EC.

Animal model and rumen digesta sample collection have previously been published by our group^5,8^ respectively.

In brief, this experiment was conducted as part of a larger study designed to examine the physiological and molecular control of compensatory growth in growing beef cattle^8^. Animals were managed on the same farm from two weeks of age prior to being transferred to Teagasc Grange Beef Research Centre, Dunsany, Co. Meath, Ireland. In order to acclimatise the animals to their environment and reduce any latent influence of previous environments, all animals were subjected to a 3 month common feeding period of *ad libitum* grass silage plus 2 kg of concentrate per head per day prior to commencing the experiment. Sixty purebred Holstein Friesian bulls (mean live weight 370 ± 35 kg; mean age 479 ± 15 d) were blocked on the basis of live weight, age and sire into four groups with fifteen animals in each group. Two of these groups received a restricted dietary allowance for 125 days (groups R and RA), with the other two groups (groups A and AA) receiving feed *ad libitum* during the same time. This 125 day period of differential feeding was followed by a subsequent *ad libitum* re-alimentation period for RA and AA groups which lasted 55 days. Throughout the 180 day trial all animals received the same total mixed ration diet consisting of 70:30 concentrate:forage (grass silage). Further details of the diet employed are provided by Keogh *et al*.^8^. The 55 d *ad libitum* period was inclusive of a 15 d transition period in order to build up R group animals to *ad libitum* feed intake. This transition period was implemented to allow animals to acclimatise to a higher plane of nutrition while preventing the development of intestinal disorders, such as acidosis. R animals were managed to grow at a rate of 0.6 kg /d, with A animals expected to gain in excess of 1.5 kg/d during the first 125 d period.

Groups R and A were slaughtered after 125 days of feeding, whilst groups RA and AA were slaughtered after the subsequent 55 day *ad libitum* re-alimentation period (following 180 days of feeding). All animals were slaughtered in an EU licensed abattoir (Euro Farm Foods Ltd, Cooksgrove, Duleek, Co. Meath, Ireland). Slaughter order was randomized to account for potential confounding effects on treatment outcomes. Two A group animals, two AA group and one R group were removed from this experiment due to illness. Two samples, one from the A group and one from the R group, were mislaid. This left 12 A, 14AA, 13 R and 15RA samples.

### Sample Collection at Slaughter

Digesta contents were sampled from five different locations, inclusive of the dorsal and ventral sacs, within the rumen of each bull immediately after slaughter. Liquid and solid fractions were separated by squeezing rumen contents through 4 layers of cheese cloth and immediately frozen in liquid nitrogen, transported on dry ice, and then stored at −80 °C.

### RNA Extraction and cDNA Synthesis

Approximately 20 g of frozen rumen liquid sample from each of the animals was considered as representative. Each sample was homogenised to a fine frozen powder under liquid nitrogen using a pestle and mortar and stored at −80 °C.

Approximately 60 mg of the homogenized frozen powder was used for RNA extraction of liquid digesta sample. RNA was extracted using the Qiagen RNeasy plus kit (Qiagen, Manchester, UK). RNA extract yield and purity were assessed with two consecutive readings on the Nanodrop 1000 spectrophotometer. RNA integrity was estimated with an Agilent 2100 Bioanalyzer (Agilent Technologies, Santa Clara, CA). Total RNA (6 µg) was DNase treated using a TURBO DNA-*free*™ Kit (Thermo Fisher Scientific, MA, USA) to remove genomic DNA. DNase treated RNA was then purified with Zymo RNA clean and concentrate kit (Zymo Research Corp, Irvine, CA, USA). The RNA concentration in each sample was normalised to 50 ng/µL and then reverse transcribed into cDNA using random hexamers and a High Capacity cDNA Reverse Transcription Kit (Thermo Fisher Scientific, MA, USA).

### *mcrA* Amplicon Library Preparation

*mcrA* amplicon libraries (*n* = 54) were generated by PCR amplification using 2.5 ng cDNA as a template and Mlas forward/ *mcrA* primers reverse^28^ fused with Nextera XT overhang adapters (Illumina, San Diego, CA, USA). PCR was performed using 1 × KAPA HiFi HotStart ReadyMix DNA polymerase (Roche Diagnositics, West Sussex, UK) and cycle conditions were initial denaturation at 95 °C for 3 minutes, then 35 amplification cycles (95 °C for 30 seconds, 55 °C for 30 seconds, 72 °C for 30 seconds) then a final extension at 72 °C for 5 minutes.

Amplicons were subsequently purified using a QIAquick PCR Purification Kit (Qiagen, Manchester, UK). A second PCR step attached dual indices and Illumina sequencing adapters using the Nextera XT index kit (Illumina, San Diego, CA, USA). Cycle conditions were an initial denaturation at 95 °C for 3 minutes, then 8 amplification cycles (95 °C for 30 seconds, 55 °C for 30 seconds, 72 °C for 30 seconds) followed by a final extension at 72 °C for 5 minutes. Amplicons were analysed on a 2% (w/v) agarose gel to check they were the correct size then pooled in equal concentrations. Pooled amplicons were gel purified to remove unwanted products using the Qiagen Gel Extraction Kit (Qiagen, Manchester, UK). An extra purification with the QIAquick purification kit was used to remove residual agarose. The pooled purified libraries were measured for purity and quantity on a Nanodrop 1000 and further quantified using the KAPA SYBR FAST universal kit with Illumina Primer Premix (Roche Diagnositics, West Sussex, UK). The library pool was then diluted and denatured as recommended by the Illumina MiSeq library preparation guide (Illumina, San Diego, CA, USA). Sequencing was conducted using 500 cycle MiSeq reagent kits (Illumina, San Diego, CA, USA).

### Sequence analysis

Read 1 and Read 2 of *mcrA* amplicons were quality checked using FASTQC^29^ (http://www.bioinformatics.babraham.ac.uk/projects/fastqc/) and overlapping reads were merged using BBMerge from the BBMap package (https://sourceforge.net/projects/bbmap/). Sequences were then size selected based on predicted amplicon fragment size (472 bp + /−1). Chimeras were removed using both a *de novo* method and against the *mcrA* database from the Functional Gene pipeline & repository (http://fungene.cme.msu.edu/). This resulted in 3,526,425 total reads from samples.

Each read was translated in all six reading frames in an effort to identify intact open reading frames. For each sequence read, a single reading frame was obtained with an expected size of ~155–160 amino acids in length and contained the conserved translation of the forward and reverse sequencing primers. This resulted in 3,526,425 MCRA peptide sequences.

Peptide sequences were clustered at 97% identity. MCRA clusters were required to account for at least 0.1% of the total peptide sequences and clusters below this threshold were discarded. This resulted in 25 MCRA clusters accounting for 96.2% of the total peptide sequences.

### Evolutionary Analysis

A representative amino acid sequence from each cluster was obtained. MCRA protein sequences from sequenced methanogen genomes were downloaded from the NCBI database (https://www.ncbi.nlm.nih.gov/protein). Genome sequences with known taxonomy were also downloaded from the *mcrA* reference database^30^ and translated into MCRA protein sequences. A database of 67 MCRA protein sequences was compiled consisting of the downloaded and representative cluster sequences. Evolutionary history was inferred using the maximum parsimony method using MEGA7.

### Statistical Analysis

Relative abundance of MCRA clusters was computed using raw sequence counts per cluster. MCRA clusters were analysed using a Kruskal-Wallis test with correction for multiple testing using the Benjamini Hochberg (BH) method to identify MCRA clusters differently represented across treatment groups. A transformation based principal coordinate analysis (PCoA) and non-metric multidimensional scaling (nMDS) was performed by square root transformation of the relative abundance data and subjecting Bray Curtis similarity analysis and then plotted using PRIMER v7 software. A heatmap was generated using PRIMER v7 software and the square root transformed relative abundance data for all four treatments. Alpha diversity (observed cluster, richness, Shannon and Simpson diversity indices) was calculated using PRIMER v7 software. Significance of alpha diversity was tested with a one-way ANOVA in R, multiple comparison analysis was assessed between treatments using the Tukey Honest Significance Difference (HSD) test in R.
